# Effects of hyperbaric oxygen combined cabin ventilator on critically ill patients with liberation difficulty after tracheostomy

**DOI:** 10.1186/s12938-024-01220-4

**Published:** 2024-03-07

**Authors:** Yinliang Qi, Jixiang Xu, Hui Liu, Xiaomei Zhou

**Affiliations:** General Department of Hyperbaric Oxygen, the Second People’s Hospital of Hefei, Hefei Affiliated Hospital of Anhui Medical University, Hefei, 230011 Anhui China

**Keywords:** Hyperbaric oxygen combined cabin ventilator, Liberation difficulty, Glasgow Coma Scale, Blood gas, Cardiac function, Clinical trial

## Abstract

**Background:**

Critically ill patients undergoing liberation often encounter various physiological and clinical complexities and challenges. However, whether the combination of hyperbaric oxygen and in-cabin ventilator therapy could offer a comprehensive approach that may simultaneously address respiratory and potentially improve outcomes in this challenging patient population remain unclear.

**Methods:**

This retrospective study involved 148 patients experiencing difficulty in liberation after tracheotomy. Inclusion criteria comprised ongoing mechanical ventilation need, lung inflammation on computed tomography (CT) scans, and Glasgow Coma Scale (GCS) scores of ≤ 9. Exclusion criteria excluded patients with active bleeding, untreated pneumothorax, cerebrospinal fluid leakage, and a heart rate below 50 beats per minute. Following exclusions, 111 cases were treated with hyperbaric oxygen combined cabin ventilator, of which 72 cases were successfully liberated (SL group) and 28 cases (NSL group) were not successfully liberated. The hyperbaric oxygen chamber group received pressurization to 0.20 MPa (2.0 ATA) for 20 min, followed by 60 min of ventilator oxygen inhalation. Successful liberation was determined by a strict process, including subjective and objective criteria, with a prolonged spontaneous breathing trial. GCS assessments were conducted to evaluate consciousness levels, with scores categorized as normal, mildly impaired, moderately impaired, or severely impaired.

**Results:**

Patients who underwent treatment exhibited improved GCS, blood gas indicators, and cardiac function indexes. The improvement of GCS, partial pressure of oxygen (PaO2), oxygen saturation of blood (SaO2), oxygenation index (OI) in the SL group was significantly higher than that of the NSL group. However, there was no significant difference in the improvement of left ventricular ejection fraction (LVEF), left ventricular end-systolic volume (LVESV), left ventricular end-diastolic volume (LVEDV), and stroke volume (SV) between the SL group and the NSL group after treatment.

**Conclusions:**

Hyperbaric oxygen combined with in-cabin ventilator therapy effectively enhances respiratory function, cardiopulmonary function, and various indicators of critically ill patients with liberation difficulty after tracheostomy.

**Supplementary Information:**

The online version contains supplementary material available at 10.1186/s12938-024-01220-4.

## Introduction

Mechanical ventilation stands as a prevalent supportive intervention for critically ill patients within the confines of the Intensive Care Unit (ICU). This method serves to rectify or mitigate acute and chronic respiratory failure stemming from diverse etiologies [1]. Nevertheless, prolonged reliance on mechanical ventilation may inflict harm upon the upper respiratory tract or induce the diaphragm atrophy. This, in turn, precipitates adverse consequences such as ventilator-associated diaphragmatic dysfunction, posing challenges to the liberation process [2]. Moreover, protracted tracheal intubation during mechanical ventilation heightens the susceptibility to pulmonary infections and exacerbates organ impairments [3]. Clinical investigations have additionally discerned that extended ventilator use hampers blood circulation in immobilized patients, fostering thrombosis and engendering pulmonary embolism [4]. Therefore, as the primary disease improves, respiratory failure is corrected, and spontaneous breathing is restored, patients should start liberation as soon as possible [5]. Delayed liberation will increase the complications of mechanical ventilation (pneumonia, airway injury, etc.), hospital stay and medical treatment cost [6].

Concomitantly, with the advancement of rigorous rehabilitation practices, the clinical efficacy of hyperbaric oxygen therapy in conditions like brain trauma, cerebral hemorrhage, and hypoxic–ischemic encephalopathy has garnered empirical confirmation [7–9]. Hyperbaric oxygen therapy involves breathing pure oxygen in a pressurized room or chamber [9]. This treatment boosts the amount of oxygen the blood can carry, promoting faster healing and reducing inflammation [10]. In addition, studies have shown that hyperbaric oxygen can increase the body's blood oxygen partial pressure, improve myocardial microcirculation, increase myocardial contractility, and increase cardiac ejection fraction [11].

To address the challenges of long-term mechanical ventilation liberation, clinical interventions typically focus on the reasons for liberation failure [12]. Some main factors contributing to liberation difficulties include inadequate control of the primary disease, poor nutritional status among long-term ventilator-dependent patients, and erroneous judgment by doctors, leading to difficulties in the liberation process [13–15]. In view of the reasons for the difficulty of liberation, conventional interventions can improve the patient's condition by controlling the primary disease, correcting the nutritional status, psychological intervention and choosing the right time for liberation [16]. However, a large number of practices have also shown that conventional intervention methods are not satisfactory for some patients. Therefore, in order to improve the success rate of liberation, more effective intervention methods are advocated [17]. Therefore, we hypothesize that the combination of hyperbaric oxygen and in-cabin ventilator therapy may have benefits for critically ill patients with liberation difficulty after tracheostomy.

## Results

### Demographic and clinical characteristics of the study subjects

**Fig. 1:**
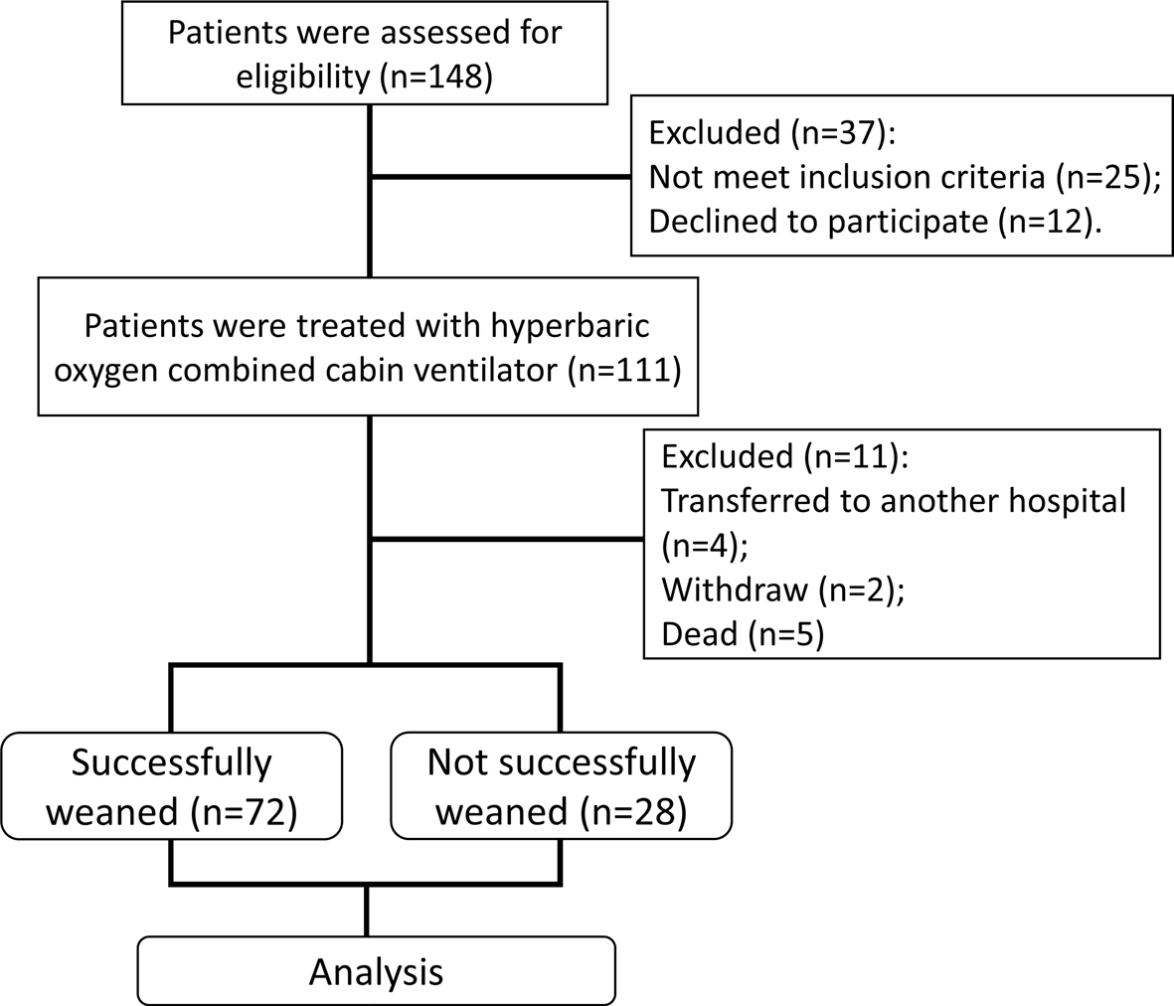
Flowchart of the work

The flowchart of the study is shown in Fig. 1. In this study, 148 patients with difficulty in liberation (after tracheotomy) were initially selected as the research subjects and were assessed for eligibility. Next, 37 patients were excluded, including those who did not meet the inclusion criteria and (*n* = 25) and those who declined to participate (*n* = 12). The remaining 111 patients were treated with hyperbaric oxygen combined with in-cabin ventilator therapy. Subsequently, 11 patients were excluded, including those who transferred to another hospital (*n* = 4), withdrew (*n* = 2), and died (*n* = 5). Finally, 72 cases were successfully liberated and 28 cases were unsuccessful liberated, and they were devoted into two groups and used for comparative analysis. Table 1 shows the demographic characteristics, primary disease status, and the statistics of the number of invasive ventilators in the hyperbaric combined cabin between the successfully liberated patients (SL group) and the unsuccessful liberation patients (NSL group). The average age of the SL group was 71.2 ± 8.8. The average age of the NSL group was 73.5 ± 9.4. Primary diseases include hypoxic–ischemic encephalopathy, respiratory failure, cerebral trauma, cerebral hemorrhage, carbon monoxide poisoning, and spinal cord injury. There was no significant difference between gender (*p* = 0.643), age (*p* = 0.118), mechanical ventilation duration (*p* = 0.336) and primary diseases (*p* = 0.355) between two groups. However, according to the ventilator liberation procedure, the number of invasive ventilators in hyperbaric oxygen combined cabin was significantly different between the two groups (*p* = 0.029).

**Table 1:** Demographic and clinical characteristics of patients who were successfully liberated (SL) and not successfully liberated (NSL)

Characteristics	Study group		*p*
	NSL (*n* = 28)	SL (*n* = 72)	
Gender			
Male	17 (60.7%)	48 (66.7%)	0.643
Female	11 (39.3%)	24 (33.3%)	
Age (years)	73.5 ± 9.4	71.2 ± 8.8	0.118
Mechanical ventilation duration (day)	15.3 ± 4.6	14.8 ± 5.1	0.336
Primary disease			
Hypoxic–ischemic encephalopathy	8 (28.6%)	10 (13.9%)	0.355
Respiratory failure	9 (32.2%)	16 (22.2%)	
Cerebral trauma	4 (14.3%)	14 (19.4%)	
Cerebral hemorrhage	3 (10.7%)	13 (18.1%)	
Carbon monoxide poisoning	2 (7.1%)	8 (11.1%)	
Spinal cord injury	2 (7.1%)	11 (15.3%)	
Number of invasive ventilators in hyperbaric oxygen combined cabin			
1–5	6 (21.4%)	38 (52.8%)	0.029
6–10	8 (28.6%)	16 (22.2%)	
11–20	10 (35.7%)	14 (19.4%)	
> 20	4 (14.3%)	4 (5.6%)	

Values were expressed as n (percentage, %) or mean ± SD. *p* values for each group were derived from Mann–Whitney test. Chi-square test or Fisher’s exact test was used for assessing distribution of observations or phenomena between two groups

### Glasgow Coma Scale (GCS) scores in the NSL and SL group

**Fig. 2:**
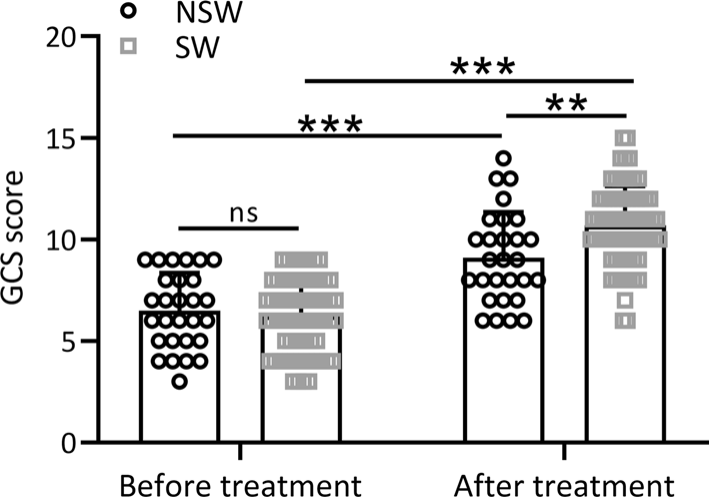
Comparisons of the GCS score between the two groups before and after hyperbaric oxygen combined cabin ventilator treatment. Data were presented as mean ± SD showing all the data points. **p < 0.01, ***p < 0.001 and ns means no significance. Two-way ANOVA followed Turkey’s multiple comparisons test

Our research findings revealed no statistically significant difference in GCS scores between the NSL and SL groups prior to treatment (*p* > 0.05, as illustrated in Fig. 2). However, following the administration of hyperbaric oxygen in conjunction with in-cabin ventilator therapy, both the SL and NSL groups exhibited noteworthy enhancements in GCS scores (*p* < 0.0001). Notably, the magnitude of improvement in GCS scores was significantly greater in the SL group compared to the NSL group (*p* = 0.0015). Additional file 1: Figure S1 visually depicts the alterations in GCS scores for all patients both before and after the treatment protocol. In summary, patients subjected to hyperbaric oxygen therapy combined with in-cabin mechanical ventilation demonstrated substantial amelioration in GCS scores, exhibiting a statistically significant difference (p < 0.0001).

### Partial pressure of oxygen (PaO2), oxygen saturation of blood (SaO2), and oxygenation index (OI) in the NSL and SL group

**Fig. 3:**
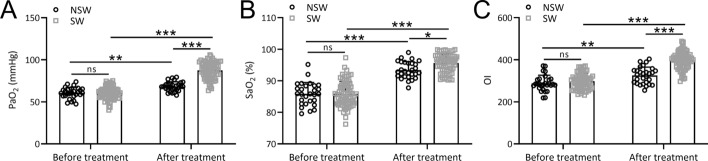
Comparisons of the PaO_2_ (**A**), SaO_2_ (**B**) and OI (**C**) between the two groups before and after hyperbaric oxygen combined cabin ventilator treatment. Data were presented as mean ± SD showing all the data points. *p < 0.05, **p < 0.01, ***p < 0.001 and ns means no significance. Two-way ANOVA followed Turkey’s multiple comparisons test

Figure 3 presents key indicators of blood gas analysis, including PaO2, SaO2, and OI. Our study results indicated no significant difference in PaO2, SaO2, and OI between the NSL and SL groups before treatment (p > 0.05, Fig. 3A–C). However, after administering hyperbaric oxygen combined with in-cabin ventilator therapy, both the SL and NSL groups demonstrated significant improvements in PaO2 (Fig. 3A), SaO2 (Fig. 3B), and OI (Fig. 3C). Notably, the improvements were significantly higher in the SL group compared to the NSL group (*p* < 0.0001). Additional file 1: Figure S2 provides a comparison of blood gas analysis for all patients before and after treatment. Overall, the PaO2, SaO2, and OI levels in patients showed significant increases after treatment (*p* < 0.0001), indicating that hyperbaric oxygen combined with in-cabin ventilator therapy effectively improved blood gas indicators in patients.

### Cardiac function indexes in the NSL and SL group

**Fig. 4:**
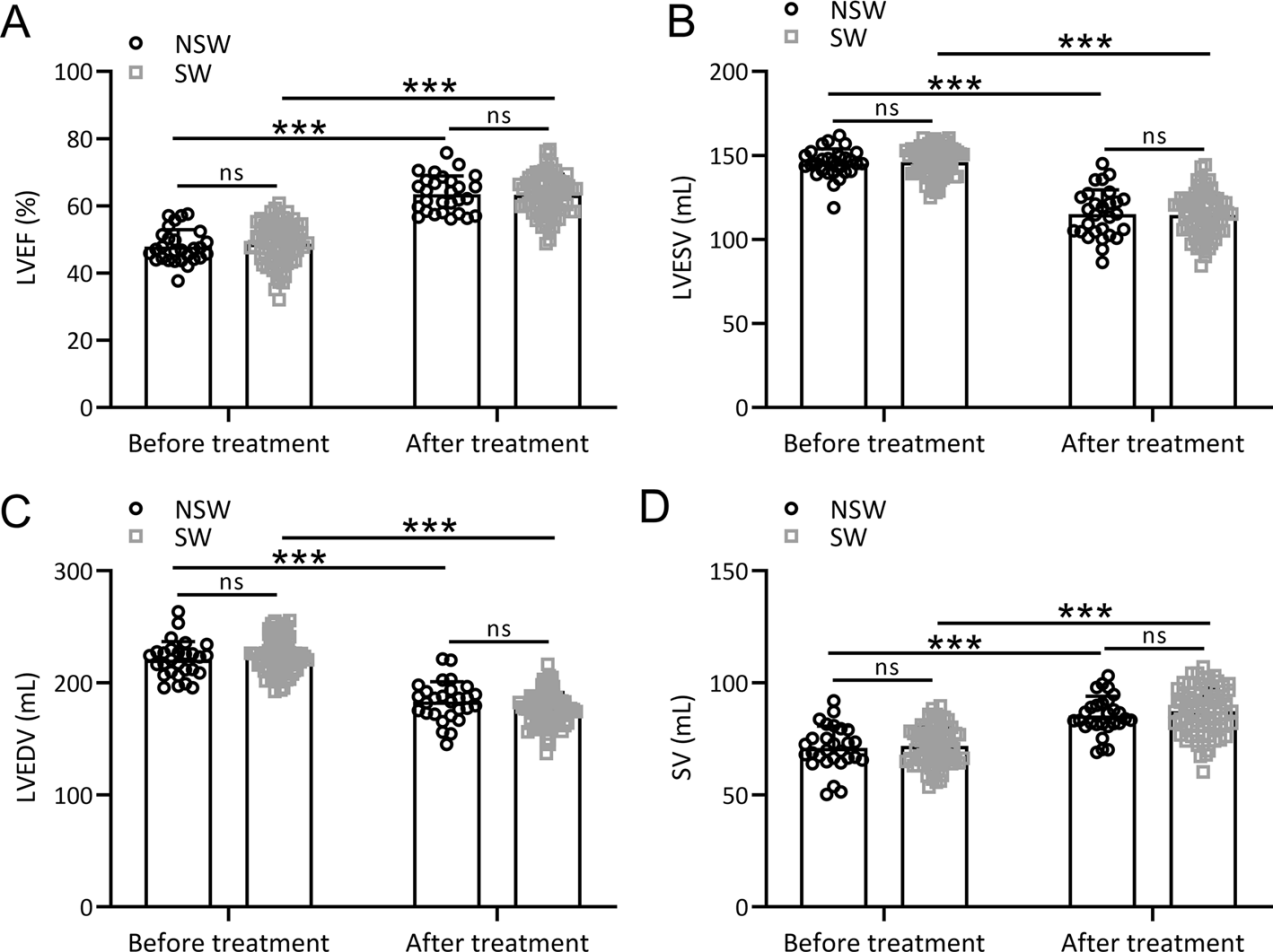
Comparisons of the LVEF (**A**), LVESV (**B**), LVEDV (**C**) and SV (**D**) between the two groups before and after hyperbaric oxygen combined cabin ventilator treatment. Data were presented as mean ± SD showing all the data points. ***p < 0.001 and ns means no significance. Two-way ANOVA followed Turkey’s multiple comparisons test

We then examined the changes in cardiac function indexes between the SL group and the NSL group. Although cardiac function is not typically used as a parameter for liberation, it still could be a potential limitation for successful liberation. Our findings revealed no significant difference in left ventricular ejection fraction (LVEF), left ventricular end-systolic volume (LVESV), left ventricular end-diastolic volume (LVEDV), and stroke volume (SV) between the NSL and SL groups before treatment (*p* > 0.05, Fig. 4A–D). However, after administering hyperbaric oxygen combined with in-cabin ventilator therapy, both the SL and NSL groups exhibited significant improvements in LVEF (Fig. 4A), LVESV (Fig. 4B), LVEDV (Fig. 4C), and SV (Fig. 4D). Nonetheless, there were no significant differences in the degree of improvement in these various cardiac function indexes between the SL and NSL groups after treatment (*p* > 0.05, Fig. 4A–D). Additional file 1: Figure S3 provides a comparison of the changes in cardiac function for all patients before and after treatment. Overall, patients showed significant increases in LVEF, LVESV, LVEDV, and SV after treatment (*p* < 0.0001), indicating that hyperbaric oxygen combined with in-cabin ventilator therapy has a significant positive impact on improving heart function indicators.

## Discussion

While mechanical ventilation plays a crucial role in emergency resuscitation and the care of critically ill patients, liberated patients off the ventilator poses a significant challenge for physicians [18]. Early initiation and prompt liberation are generally recommended for mechanically ventilated patients. However, difficulties in liberation can lead to complications, impair respiratory function, and increase the financial burden on patients [19].

At present, hyperbaric oxygen therapy has been widely used in the treatment of cerebrovascular diseases and other diseases [20]. Early implementation of hyperbaric oxygen therapy promotes the growth rate of nerve cell axons, facilitates the development of collateral cerebral circulation, enhances compensation and reorganization of healthy brain cells or tissues around the lesion, improves brain plasticity, and supports effective recovery of brain function [21]. Administering in-cabin hyperbaric oxygen treatment promptly not only improves hypoxia and reduces intracranial pressure, but also mitigates nerve cell apoptosis and lowers the disability rate [22]. The application of an in-cabin ventilator, in conjunction with hyperbaric oxygen therapy, ensures patients can maintain stable physical signs within the unique hyperbaric oxygen environment. It also provides auxiliary support for successful liberation [22].

The study began with 148 patients facing challenges in liberation after tracheotomy, ultimately resulting in 72 successfully liberated cases and 28 unsuccessful cases. A comprehensive analysis of demographic characteristics, primary disease status, and the number of invasive ventilators revealed no significant differences in gender, age, mechanical ventilation duration, and primary diseases between the SL and NSL groups. However, the number of invasive ventilators in the hyperbaric oxygen combined cabin was notably different between the two groups (*p* = 0.029). This result indicates that too many times of invasive ventilator may prevent successful liberation of the patients treated with hyperbaric oxygen. Prior to treatment, there were no significant differences in GCS scores between the NSL and SL groups. Following hyperbaric oxygen therapy combined with in-cabin ventilator therapy, both groups exhibited substantial improvements in GCS scores, with the SL group demonstrating a significantly greater enhancement compared to the NSL group (*p* = 0.0015). This outcome underscores the efficacy of the combined therapy in enhancing neurological outcomes, with clinical implications for patients undergoing liberation difficulties after tracheotomy.

Blood gas analysis indicators, including PaO2, SaO2, and OI, demonstrated no significant differences between the NSL and SL groups before treatment. However, post-treatment, both groups exhibited significant improvements in these indicators, with the SL group showing significantly greater enhancement. This suggests that hyperbaric oxygen combined with in-cabin ventilator therapy effectively improved blood gas parameters, highlighting its clinical utility in optimizing oxygenation in patients with liberation difficulties. Initial assessments revealed no significant differences in cardiac function indexes between the NSL and SL groups. After treatment, both groups exhibited significant improvements in LVEF, LVESV, LVEDV, and SV. Importantly, no significant differences were observed in the degree of improvement between the two groups. These results indicate a positive impact of hyperbaric oxygen combined with in-cabin ventilator therapy on cardiac function, emphasizing its potential benefits in enhancing heart function indicators.

Indeed, there are certain limitations of the study. The study included a total of 111 patients after exclusion criteria, which may be considered a relatively small sample size. The results could be due to significant bias. A larger sample size would provide more robust statistical power and increase the generalizability of the findings. Secondly, the study selected patients from a specific hospital, which may introduce selection bias and limit the generalizability of the results to other settings or patient populations. The inclusion criteria and exclusion criteria used in the study may have also influenced the characteristics of the patient sample. Thirdly, the study primarily focuses on the effects of hyperbaric oxygen combined with in-cabin ventilator therapy on liberation difficulty. However, there is no mention of long-term follow-up to assess the durability of the observed outcomes or the potential for relapse after treatment. In addition, the main reason for patients' improvement in GCS is symptomatic surgery and treatment, and hyperbaric oxygen may be only a small reason. Since our study is a retrospective study, we can no longer compare the GCS of patients treated with hyperbaric oxygen and those without treatment.

## Conclusion

In summary, the comprehensive analysis of the study results supports the clinical use of hyperbaric oxygen therapy combined with in-cabin mechanical ventilation in patients facing liberation difficulties after tracheotomy. The combined therapy demonstrates positive effects on neurological outcomes, blood gas parameters, and cardiac function, underscoring its potential as a valuable intervention in clinical practice. However, prospective data are needed to further demonstrate the benefit of the combined therapy.

## Methods

### Subjects

This is a retrospective study. In this study, 148 patients with difficulty in liberation (after tracheotomy) who were admitted to the hyperbaric oxygen department of the Second People’s Hospital of Hefei, Hefei Affiliated Hospital of Anhui Medical University from January 1, 2018 to December 31, 2020 were selected as the research objects.

Inclusion criteria: Patients admitted to the hospital with an ongoing need for mechanical ventilation, exhibiting lung inflammation on computed tomography (CT) scans, and having Glasgow Coma Scale (GCS) scores of ≤ 9 points.

Exclusion criteria: Patients with active bleeding, untreated pneumothorax, cerebrospinal fluid leakage, and a heart rate below 50 beats per minute.

After exclusion, 111 cases were treated with hyperbaric oxygen combined cabin ventilator, 11 cases were lost midway, 72 cases were successfully liberated, and 28 cases were not successfully liberated. The detailed process is shown in Fig. 1. The study was approved by the ethics committee of Second People’s Hospital of Hefei, Hefei Affiliated Hospital of Anhui Medical University (2022-Scientific Research-091).

### Treatment programs

All patients were ventilated with a hyperbaric oxygen combined cabin ventilator, using a domestically made hyperbaric cabin dedicated AII6000B plus ventilator. Ventilation mode adopts SIMV mode, trigger sensitivity: −2 cm H2O; working pressure: ambient pressure + 0.60 MPa.

Treatment plan: Three-cabin and seven-door hyperbaric oxygen chamber group is adopted, air is pressurized, the treatment pressure is 0.20 MPa (2.0 ATA), the pressurization time is 20 min, the oxygen inhalation time of the ventilator is 60 min. Among them, the rest was 10 min, the decompression time was 20 min, the total treatment time was about 110 min, and the treatment was performed once a day. Medical staff will accompany the cabin throughout the treatment.

Other treatments: Dehydration, anti-infection, nourishment of nerves, expansion of blood vessels, improvement of microcirculation, nutritional support, anti-oxygen free radicals, rehabilitation and other treatments are given at different stages of the disease course.

### Judgment of successful liberation of patients with mechanical ventilation

This study strictly implemented the ventilator liberation process. First of all, from a subjective and objective perspective, patients with improved conditions enter the stage of ventilator liberation screening. The subjective point of view is that the clinician believes that the cause of the spontaneous breathing has been improved or eliminated. The objective point of view is that the patient’s oxygenation index, positive end-expiratory pressure, inhaled oxygen concentration, and arterial blood pH are all within normal values; hemodynamic stability evaluation: patient's oxygen levels, respiratory parameters, arterial blood pH, stability in blood pressure, the disappearance of myocardial ischemia, and the possibility to discontinue vasoactive drugs. Following the initial ventilator liberation assessment and screening, patients proceed to undergo a spontaneous breathing trial (SBT). In this investigative cohort, the SBT procedure employs a T tube for direct disconnection from the ventilator. Upon successful completion of a 3-min SBT, patients autonomously breathe for an additional 30 min. Typically, individuals capable of tolerating this trial are deemed successfully weaned. However, given the specific focus on patients grappling with liberation challenges in this study, the spontaneous breathing duration is extended. Specifically, success in liberation is determined for patients who can sustain continuous spontaneous breathing for a prolonged period of 24 h.

### Statistical analysis

Values were expressed as n (percentage, %) or mean ± SD. Four tests of Shapiro–Wilk test, Anderson–Darling test, Kolmogorov–Smirnov test, D’Agostino and Pearson test were used to test the normality of the data firstly. p values of the demographic and clinical characteristics for each group were derived from Mann–Whitney test. Chi-square test or Fisher’s exact test was used for assessing distribution of observations or phenomena between two groups. Mann–Whitney test was used when the data were not normal. Unpaired t test with Welch's correction was used when the data were normal. Two-way ANOVA followed Turkey’s multiple comparisons test was used to compare the differences between the groups with different time points. *p < 0.05, **p < 0.01, ***p < 0.001 and ns means no significance.

### Supplementary Information

**Additional file 1: Figure S1.** Comparisons of the GCS score of all the patients before and after hyperbaric oxygen combined cabin ventilator treatment. Data were presented as mean ± SD showing all the data points. ***p < 0.001. Unpaired t test with Welch's correction. **Figure S2.** Comparisons of the PaO_2_ (A), SaO_2_ (B) and OI (C) of all the patients before and after hyperbaric oxygen combined cabin ventilator treatment. Data were presented as mean ± SD showing all the data points. ***p < 0.001. Unpaired t test with Welch's correction. **Figure S3.** Comparisons of the LVEF (A), LVESV (B), LVEDV (C) and SV (C) of all the patients before and after hyperbaric oxygen combined cabin ventilator treatment. Data were presented as mean ± SD showing all the data points. ***p < 0.001. Unpaired t test with Welch's correction. **Figure S4.** Comparisons of the T4 (A), T3 (B), FT3 (C), FT4 (D) and TSH (E) of all the patients before and after hyperbaric oxygen combined cabin ventilator treatment. Data were presented as mean ± SD showing all the data points. ***p < 0.001. Unpaired t test with Welch's correction.

## Data Availability

The raw data supporting the conclusions of this article will be made available by the authors, without undue reservation.
