# Supplementary material for: Effects of hyperbaric oxygen combined cabin ventilator on critically ill patients with liberation difficulty after tracheostomy
Source: Biomed Eng Online. 2024 Mar 7;23:30. doi: 10.1186/s12938-024-01220-4 (PMC10921656; doi:10.1186/s12938-024-01220-4)
Supplement: Supplementary file 1 — Additional file 1: Figure S1. Comparisons of the GCS score of all the patients before and after hyperbaric oxygen combined cabin ventilator treatment. Data were presented as mean ± SD showing all the data points. ***p < 0.001. Unpaired t test with Welch's correction. Figure S2. Comparisons of the PaO2 (A), SaO2 (B) and OI (C) of all the patients before and after hyperbaric oxygen combined cabin ventilator treatment. Data were presented as mean ± SD showing all the data points. ***p < 0.001. Unpaired t test with Welch's correction. Figure S3. Comparisons of the LVEF (A), LVESV (B), LVEDV (C) and SV (C) of all the patients before and after hyperbaric oxygen combined cabin ventilator treatment. Data were presented as mean ± SD showing all the data points. ***p < 0.001. Unpaired t test with Welch's correction. Figure S4. Comparisons of the T4 (A), T3 (B), FT3 (C), FT4 (D) and TSH (E) of all the patients before and after hyperbaric oxygen combined cabin ventilator treatment. Data were presented as mean ± SD showing all the data points. ***p < 0.001. Unpaired t test with Welch's correction. [file 12938_2024_1220_MOESM1_ESM.docx]

**Additional file**

**
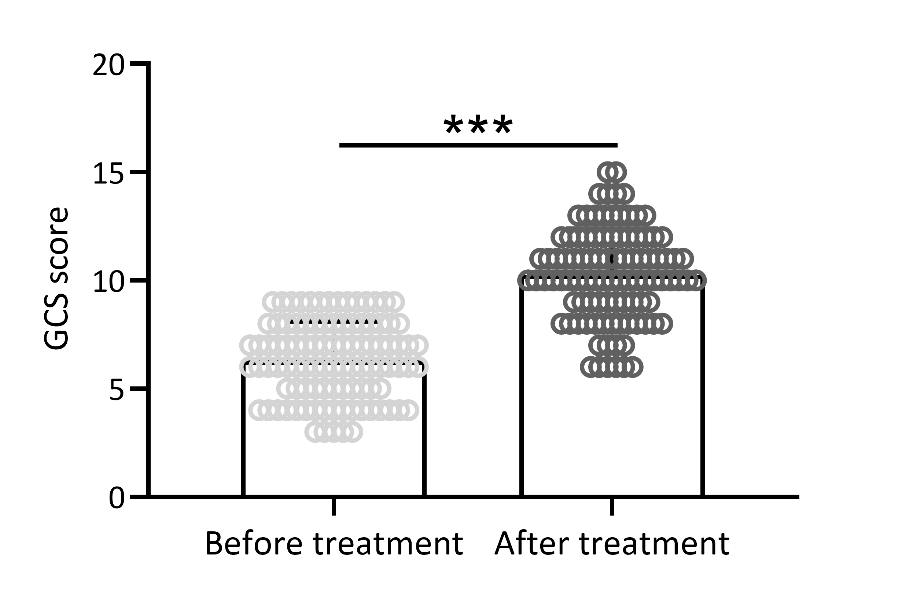
**

**Figure S1. Comparisons of the GCS score of all the patients before and after hyperbaric oxygen combined cabin ventilator treatment.** Data were presented as mean ± SD showing all the data points. ***p < 0.001. Unpaired t test with Welch's correction.


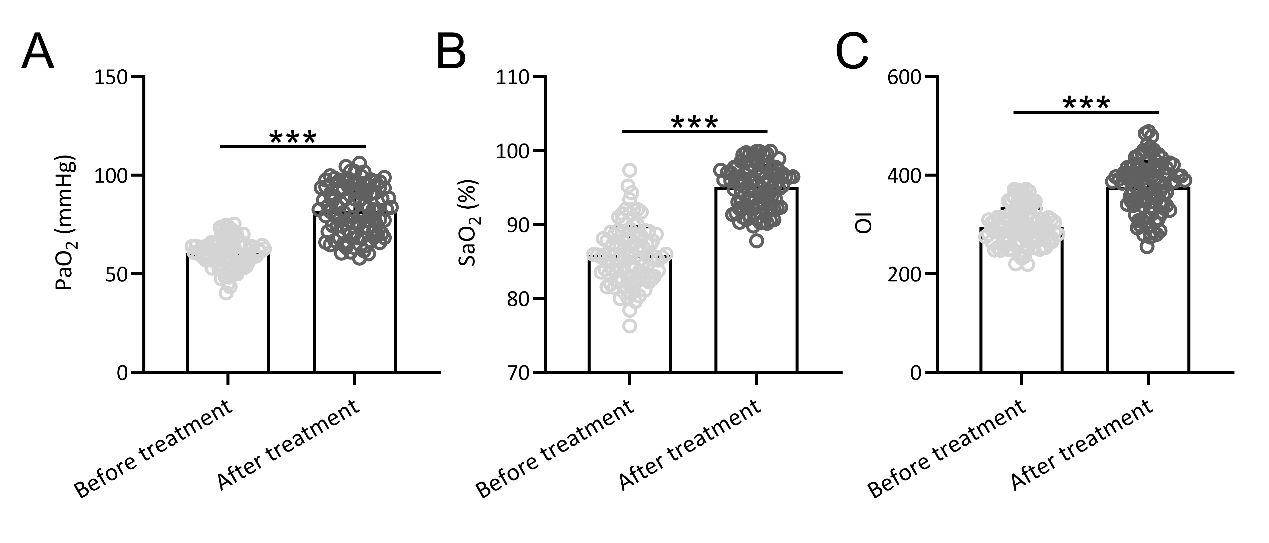


**Figure S2. Comparisons of the PaO_2_ (A), SaO_2_ (B) and OI (C) of all the patients before and after hyperbaric oxygen combined cabin ventilator treatment.** Data were presented as mean ± SD showing all the data points. ***p < 0.001. Unpaired t test with Welch's correction.


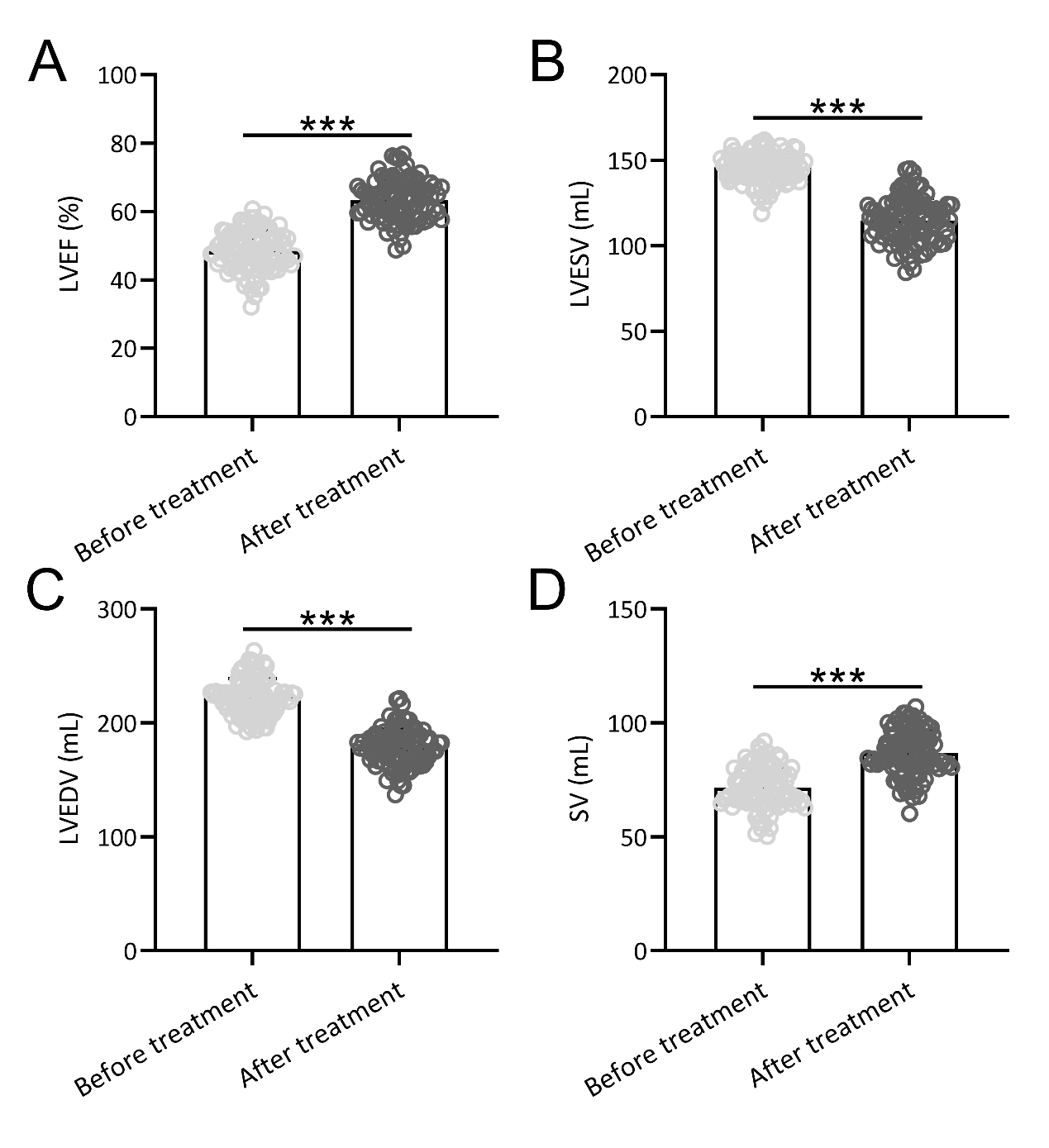


**Figure S3. Comparisons of the LVEF (A), LVESV (B), LVEDV (C) and SV (C) of all the patients before and after hyperbaric oxygen combined cabin ventilator treatment.** Data were presented as mean ± SD showing all the data points. ***p < 0.001. Unpaired t test with Welch's correction.


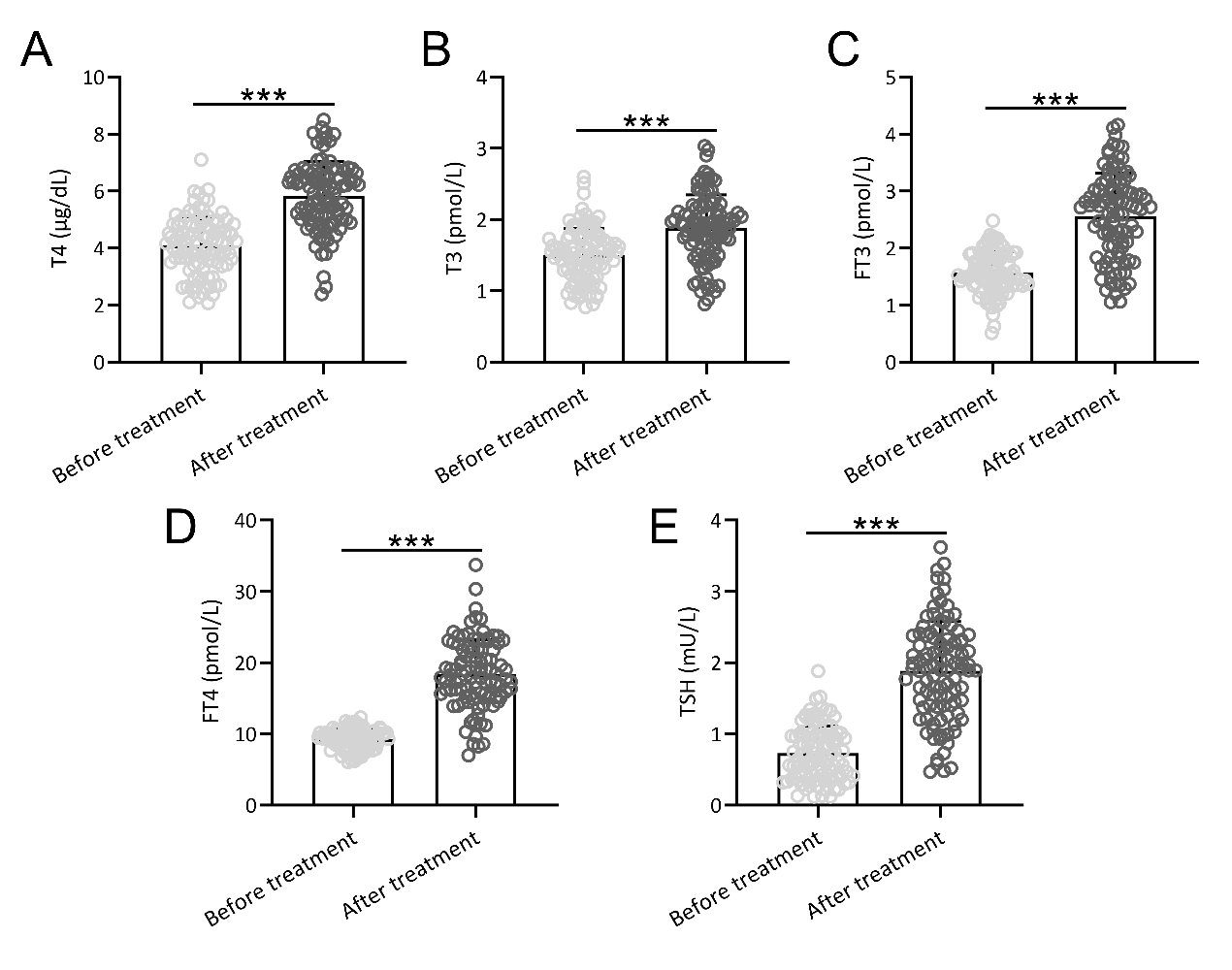


**Figure S4. Comparisons of the T4 (A), T3 (B), FT3 (C), FT4 (D) and TSH (E) of all the patients before and after hyperbaric oxygen combined cabin ventilator treatment.** Data were presented as mean ± SD showing all the data points. ***p < 0.001. Unpaired t test with Welch's correction.
